# Clinical significance of metabolism-related genes and FAK activity in ovarian high-grade serous carcinoma

**DOI:** 10.1186/s12885-021-09148-x

**Published:** 2022-01-13

**Authors:** Masakazu Sato, Sho Sato, Daisuke Shintani, Mieko Hanaoka, Aiko Ogasawara, Maiko Miwa, Akira Yabuno, Akira Kurosaki, Hiroyuki Yoshida, Keiichi Fujiwara, Kosei Hasegawa

**Affiliations:** Department of Gynecologic Oncology, Saitama Medical University International Medical Center, 1397-1 Yamane, Hidaka, Saitama, 350-1298 Japan

**Keywords:** Ovarian cancer, Platinum resistance, Machine learning

## Abstract

**Background:**

Administration of poly (ADP-ribose) polymerase (PARP) inhibitors after achieving a response to platinum-containing drugs significantly prolonged relapse-free survival compared to placebo administration. PARP inhibitors have been used in clinical practice. However, patients with platinum-resistant relapsed ovarian cancer still have a poor prognosis and there is an unmet need. The purpose of this study was to examine the clinical significance of metabolic genes and focal adhesion kinase (FAK) activity in advanced ovarian high-grade serous carcinoma (HGSC).

**Methods:**

The RNA sequencing (RNA-seq) data and clinical data of HGSC patients were obtained from the Genomic Data Commons (GDC) Data Portal and analysed (https://portal.gdc.cancer.gov/). In addition, tumour tissue was sampled by laparotomy or screening laparoscopy prior to treatment initiation from patients diagnosed with stage IIIC ovarian cancer (International Federation of Gynecology and Obstetrics (FIGO) classification, 2014) at the Saitama Medical University International Medical Center, and among the patients diagnosed with HGSC, 16 cases of available cryopreserved specimens were included in this study. The present study was reviewed and approved by the Institutional Review Board of Saitama Medical University International Medical Center (Saitama, Japan). Among the 6307 variable genes detected in both The Cancer Genome Atlas-Ovarian (TCGA-OV) data and clinical specimen data, 35 genes related to metabolism and FAK activity were applied. RNA-seq data were analysed using the Subio Platform (Subio Inc, Japan). JMP 15 (SAS, USA) was used for statistical analysis and various types of machine learning. The Kaplan-Meier method was used for survival analysis, and the Wilcoxon test was used to analyse significant differences. P < 0.05 was considered significant.

**Results:**

In the TCGA-OV data, patients with stage IIIC with a residual tumour diameter of 1-10 mm were selected for K means clustering and classified into groups with significant prognostic correlations (*p* = 0.0444). These groups were significantly associated with platinum sensitivity/resistance in clinical cases (χ^2^ test, *p* = 0.0408) and showed significant relationships with progression-free survival (*p* = 0.0307).

**Conclusion:**

In the TCGA-OV data, 2 groups classified by clustering focusing on metabolism-related genes and FAK activity were shown to be associated with platinum resistance and a poor prognosis.

**Supplementary Information:**

The online version contains supplementary material available at 10.1186/s12885-021-09148-x.

## Background

Gynecological malignancies include cervical cancer, uterine cancer, and ovarian cancer, among others. Ovarian cancer is the 5th leading cause of cancer deaths among women worldwide and is considered to have an extremely poor prognosis [1–3]. One of the reasons for the poor prognosis is that most patients are asymptomatic, and most cases are discovered at an advanced stage, i.e., with dissemination or metastasis in the abdominal cavity [4]. Although the prognosis of ovarian cancer patients has dramatically improved since the advent of paclitaxel and carboplatin combination therapy (TC therapy), the prognosis is still poor for advanced stage III and IV patients, who account for 60% of ovarian cancer patients [4–7]. One of the reasons for the poor prognosis of patients with advanced stage is the tendency for relapse. Ovarian cancer is reported to respond well to initial treatment (platinum drugs including carboplatin as mentioned above); however, approximately half of cases will relapse [1]. Since achieving a radical cure is difficult after relapse, treatment after relapse mainly aims to prolong survival and alleviate symptoms [5–9]. Thus, treatments that do not cause relapse or metastasis and treatments that provide hope for remission even after relapse/metastasis are urgently needed.

Recently, clinical trials have shown that administration of poly (ADP-ribose) polymerase (PARP) inhibitors to ovarian cancer patients after achieving a response to platinum-containing drugs significantly prolonged relapse-free survival compared to placebo administration. PARP inhibitors are used in actual clinical practice [10–17]. Thus, a promising medication has emerged for platinum-sensitive patients. However, the prognosis of platinum-resistant patients is still poor. Thus, new drugs must be developed because platinum sensitivity or platinum resistance cannot be identified without administration of a platinum-containing drug. If a method is developed to predict platinum resistance or platinum sensitivity before administration, proper treatment can be offered to each individual patient [18, 19].

The involvement of cancer stem cells (CSCs) in cancer relapse and treatment resistance has been reported in recent years, indicating that cancer tissues are heterogeneous and that some cancer cells, such as CSCs, are involved in relapse and treatment resistance [20–23]. Even if non-cancer stem cells (non-CSCs) are treated, they can lead to relapse as long as a CSC is alive. Conversely, if CSCs are eradicated, the remained cancer tissue (non-CSCs) will eventually be eliminated by host antitumor immunity. From the results of RNA sequencing (RNA-seq) and metabolomic analysis using cell lines, the authors found that the metabolic pathway and Focal adhesion kinase (FAK) activity associated with CSCs for gynecologic cancer may differ from those of non-CSCs [24].

Therefore, the purpose of this study was to examine the clinical significance of metabolic genes and FAK activity in advanced ovarian high-grade serous carcinoma (HGSC). Specifically, RNA-seq was performed on cancer specimens before treatment initiation to examine relationships with the effects of platinum-containing drugs with an emphasis on metabolic genes and FAK activity. Machine learning including cluster analysis was used for analysis.

Using machine learning, predicting prognoses for cancer patients and the therapeutic effects of platinum-containing drugs can be widely performed [25–36]. In this study, by showing that the therapeutic effect can be predicted using metabolic genes and FAK activity, these variables were confirmed to be clinically significant.

## Methods

### Patient and sample collection

The present study was reviewed and approved by the Institutional Review Board of Saitama Medical University International Medical Center (approval no.13-165). Patients diagnosed with ovarian cancer stage IIIC (International Federation of Gynecology and Obstetrics (FIGO) classification 2014) who started treatment at Saitama Medical University International Medical Center between November 2008 and August 2016 were targeted. There were 101 patients with HGSC who had stage IIIC tumours in that period, and tumour tissue sampling was performed during open surgery or exploratory laparoscopy before treatment initiation. Among them, representative 16 cases with available cryopreserved specimens were analysed.

Tumour specimens were collected by surgery and immediately cryopreserved at -80 °C. Total RNA was extracted as previously reported [37]. In brief, RNA was extracted from the frozen tissues using NucleoSpin RNA (Takara, Japan). Quality control was performed using a Bioanalyzer (Agilent, USA), and all RNA integrity number (RIN) values were > 8.0.

The clinical information of the 16 cases were obtained from the electrical health record, and is shown in Table 1. Platinum-based neoadjuvant chemotherapy (NAC) was performed as primary treatment, and an interval debulking surgery (IDS) was performed when the effect was confirmed. The Response Evaluation Criteria in Solid Tumours (RECIST) were used to determine the therapeutic effect [38]. As a guideline to measure the effect of chemotherapy, the period from administration of the last platinum-containing chemotherapy until disease deterioration (platinum-free interval, PFI) was examined [39].

**Table 1:** Clinical specimen data

Sample	Age	Treatment effect	Vital status	PFI (Months)	Clusters
1	66	PD	1	4	2
2	54	CR	1	4	2
3	59	PD	1	6	1
4	66	CR	1	0	2
5	75	SD	1	7	2
6	56	PR	1	5	2
7	70	CR	1	0	1
8	62	PR	0	0	2
9	72	PR	1	42	1
10	44	PR	0	22	1
11	70	PR	1	21	2
12	71	CR	0	34	1
13	66	PR	0	29	1
14	52	PR	0	17	1
15	66	CR	0	37	1
16	54	CR	0	24	2

Sixteen patients diagnosed with ovarian high-grade serous carcinoma (HGSC), stage IIIC (FIGO classification 2014), who had started treatment were analysed

*CR* complete response, *PR* partial response, *SD* stable disease, *PD* progressive disease

Vital status: alive = 0, dead = 1. *PFI* Platinum-free interval. Cluster: Cluster classified by cluster analysis

### RNA-seq

RNA sequencing was performed using the Illumina NovaSeq 6000 platform with a standard 100-bp paired-end read protocol as previously described [40]. Libraries for RNA-seq were prepared using the TruSeq Stranded mRNA LT Sample Prep Kit for Illumina (New England BioLabs, USA). The reference genome sequence of *Homo sapiens* (hg19) and annotation data were downloaded from the UCSC table browser (http://genome.ucsc.edu). The results of sample qualities were shown in Figs. S1, S2 and S3.

### The cancer genome atlas-ovarian (TCGA-OV)

The RNA-seq data and clinical data of ovarian cancer patients were obtained from the Genomic Data Commons (GDC) Data Portal (https://portal.gdc.cancer.gov/) [41–51].

RNA seq data for ovarian cancer patients available at TCGA were extracted on October 30, 2019. The RNA-Seq dataset consisted of 378 samples. A total of 373 primary tumour samples and 5 recurrent tumour samples were included.

### Data analysis

RNA-seq data were analysed using the Subio Platform (Subio Inc, Japan) [52].

### TCGA-OV data

The read count value data were analysed. Normalization/preprocessing was performed as follows. For log transformation, the read count value was converted to a logarithm with a base of 2. If the read count was 0, a missing value was documented. Subsequently, global normalization was performed with the 90th percentile. Then, for the low signal cutoff, if the value after normalization was less than 50, it was replaced with 50 and used as the cutoff value. To account for missing values, original read counts of 0, indicating a missing value, were assigned a value of 2 to the 5th power.

For centring, the expression level of each gene was converted to the ratio against the average value. The value generated by applying the above normalization and preprocessing is displayed as a value called the Processed Signal on the Subio Platform and is the log2 ratio against the average value of the expression levels of all samples for each gene.

Measurement values with a read count less than 100 were considered to be unreliable, and genes with a read count value less than 50 were excluded from the analysis in 189 samples, reflecting half of the 378 samples. Thus, 16,485 genes were extracted.

### Clinical specimen data

Similar to the TCGA-OV data, the clinical specimen data were normalized and preprocessed. However, the processing method is fine-tuned on the basis of sample size and the distribution of read count values.

For log transformation, the read count value was converted to a logarithm with a base of 2. However, if the read count value was 0, logarithmic transformation was not possible, and the result was replaced with a missing value. Subsequently, global normalization was performed through alignment with the 75th percentile. Then, when the value after normalization was smaller than 100 (low signal cutoff), it was replaced with 100. To account for missing values, sites with a missing value due to an original read count of 0 were assigned a value of 2 to the 6th power.

The value generated by applying the above processing is displayed as the Processed Signal on the Subio Platform as well as the TCGA-OV data. Measurement values with a read count value less than 100 were considered to be unreliable, and these genes were removed. To exclude genes whose expression did not change and genes whose expression changed randomly, genes whose average Processed Signal was in the range of -0.3 to 0.3 were removed. Thus, 6840 genes were extracted.

Finally, the Processed Signal of 6307 genes, which was extracted from TCGA-OV data and clinical sample data, was selected as a candidate of the variable to be used in the machine learning analysis. There are many genes related to FAK pathways and metabolism, however, selecting many variables for machine learning could result in overfitting [53]. And we focused on major metabolic and FAK pathway genes related to such as glycolysis, Krebs cycle, serine metabolism, glutamine metabolism and integrins [54–58].

### Statistical analysis

JMP 15 (SAS, USA) was used for statistical analysis and various types of machine learning. The Kaplan-Meier method was used for survival analysis, and the Wilcoxon test was used to analyse significant differences. *P* < 0.05 was considered significant.

## Results

### TCGA-OV data

**Fig. 1.**
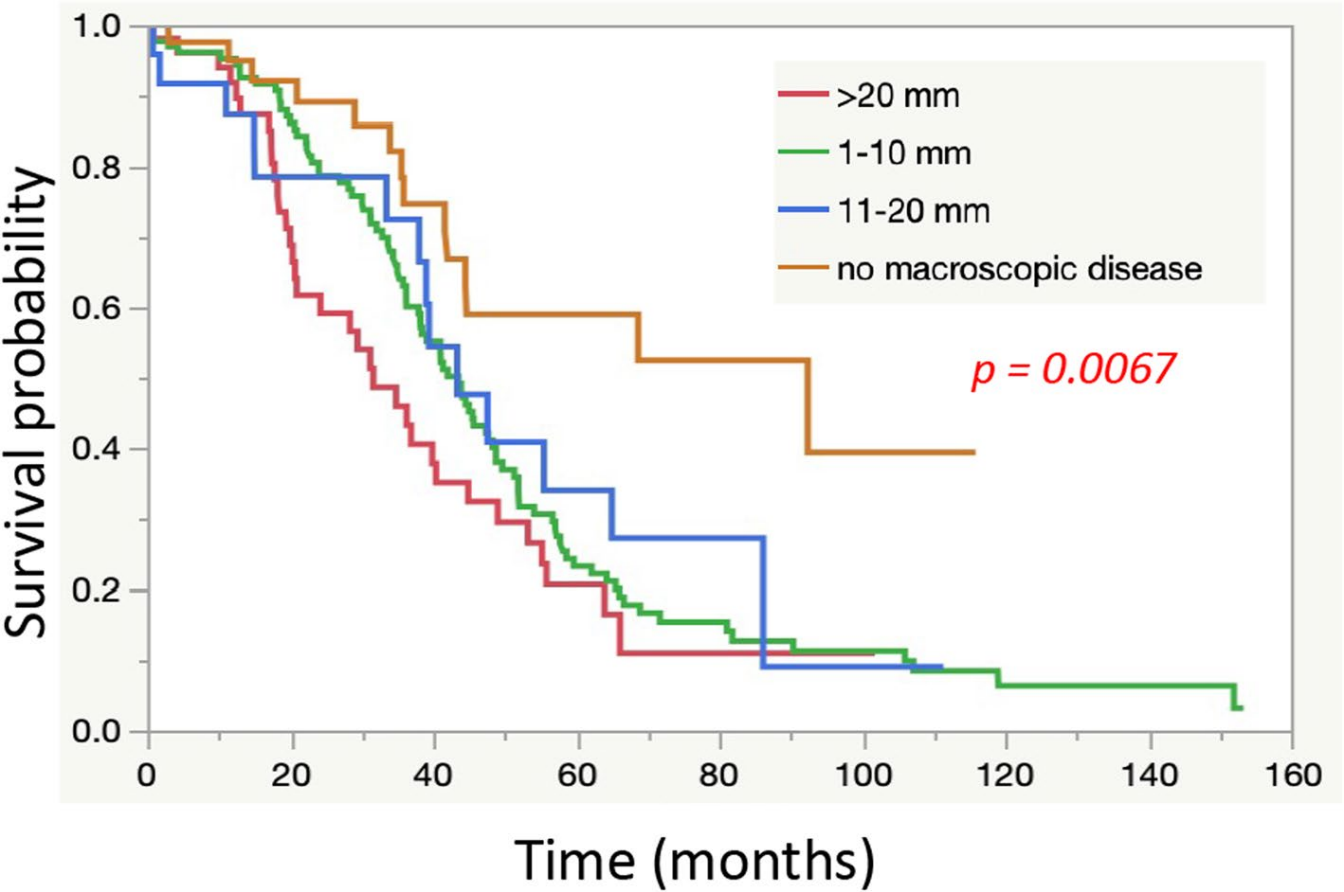
Relationship between a Residual Tumour at the Time of Surgery and the Prognosis of Patients with Advanced Stage IIIC. A larger residual tumour diameter corresponds to a worse prognosis (*p* = 0.0067)

TCGA-OV data included data from ovarian cancer patients with advanced stage I to stage IV disease, but since the prognosis differs depending on the stage of advancement, in this study, we analysed the data for the patients with stage III ovarian cancer. However, in the treatment of ovarian cancers, the prognosis differs depending on the amount of residual tumour at the time of surgery [59]. In other words, in the treatment of ovarian cancer, surgery resulting in no residual tumour is considered complete surgery with a good prognosis, while surgery resulting in a residual tumour exceeding 1 cm in diameter is considered suboptimal surgery without a good prognosis. Surgery resulting in a residual tumour with a diameter within 1-10 mm is considered optimal surgery. In practice, even in the TCGA-OV data, as shown in Fig. 1, the prognosis was poor depending on the amount of residual tumour during surgery. In other words, when considering the relationship between the prognosis and biological characteristics of cancer tissue, the results may differ depending on the residual tumour diameter. In this study, the medical case with a residual tumour measuring between 1-10 mm was extracted and analysed. The clinical information including the prognosis of 130 cases was obtained and studied.

### Classification by cluster analysis

In 130 cases obtained as described above, cluster analysis was performed for gene expression, as shown in Table 2. The selection of genes is described in the Introduction and Discussion. The genes related to metabolism and FAK activity were studied.

**Table 2:** Genes used for clustering analysis

Gene name				
CAV1	GLUD1	GOT1	GPT	GPT2
HK1	HOOK1	ITGA1	ITGA11	ITGA2
ITGA3	ITGA4	ITGA5	ITGA6	ITGA7
ITGA9	ITGAL	ITGAM	ITGAV	ITGAX
ITGB1	ITGB2	ITGB3	ITGB4	ITGB5
ITGB6	LDHA	LDHB	PHGDH	PSAT1
PSPH	ROCK2	SLC1A5	SLC7A5	SRC

Among the 6307 genes detected in both TCGA-OV data and clinical specimen data for FAK activity, 35 genes related to metabolism were analysed

**Fig. 2:**
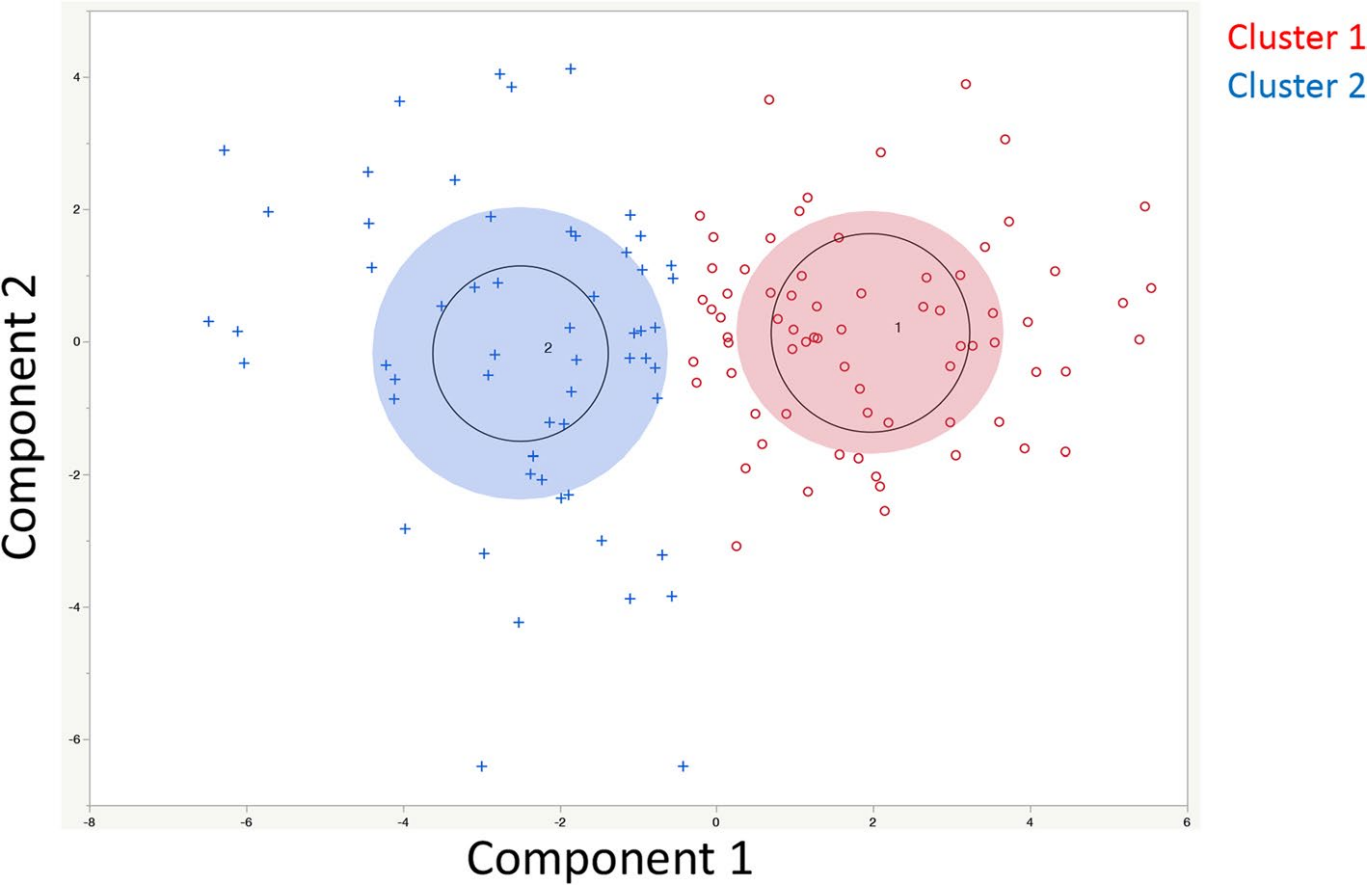
Clustering Results. Results classified by K means clustering. The 2 groups were clearly classified

**Fig. 3:**
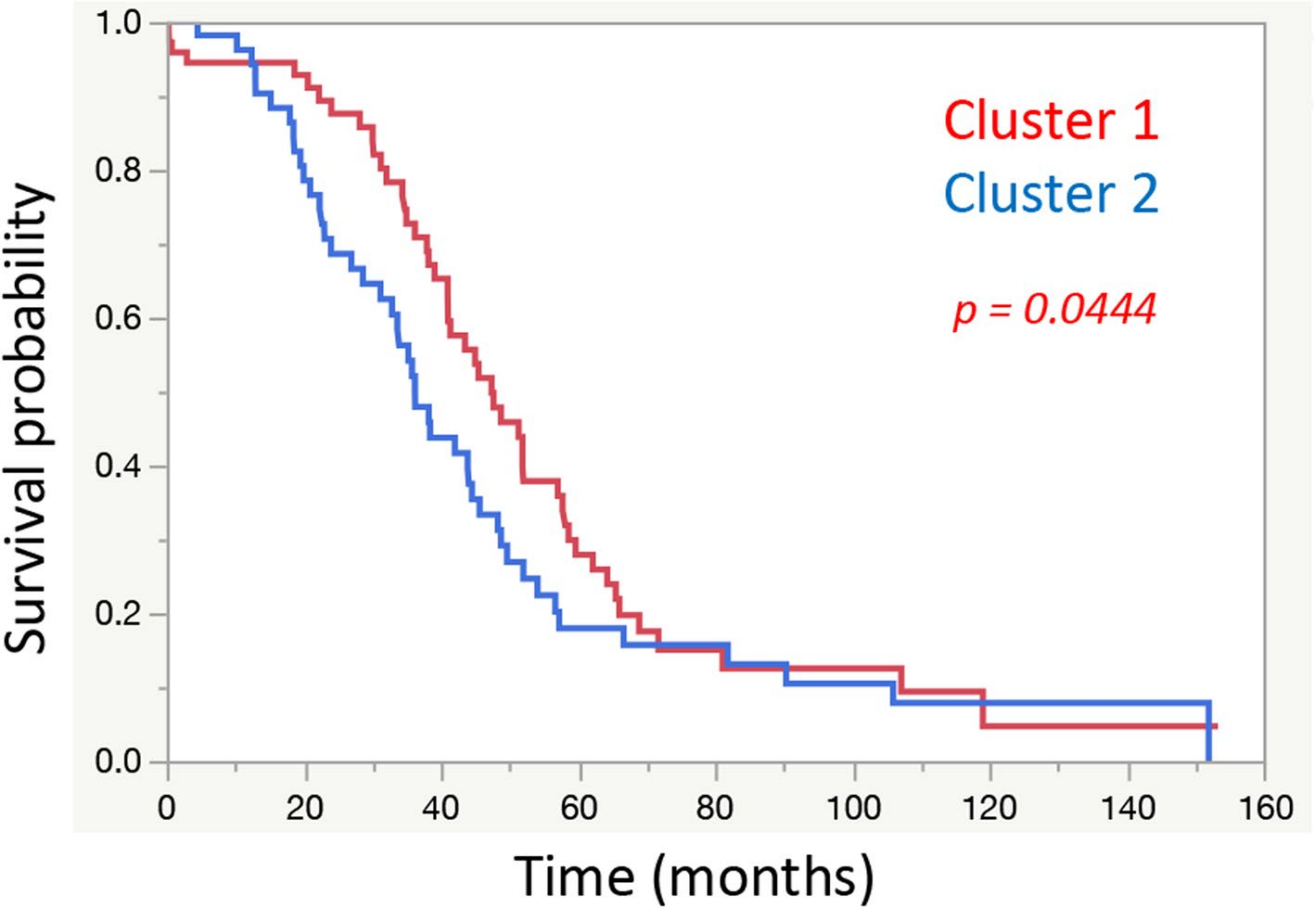
Relationships with Prognosis based on Clustering Results. Among the clusters classified by K means clustering, cluster 2 had a significantly worse prognosis than cluster 1 (*p* = 0.0444)

A total of 130 cases were classified into 2 groups (Fig. 2) according to K means clustering [60]. As shown in Fig. 3, the results were classified into 2 groups, which were significantly related to prognosis (Wilcoxon-test, *p* = 0.0444). The mean value and manifestation of each gene in these groups are shown in Fig. S4. Regarding metabolic genes, both high and low expression levels and the overall balance were involved in the metabolic phenotype [61]. Therefore, in this examination, the difference between these 2 groups was unclear.

### Analysis including clinical specimen data

**Fig. 4:**
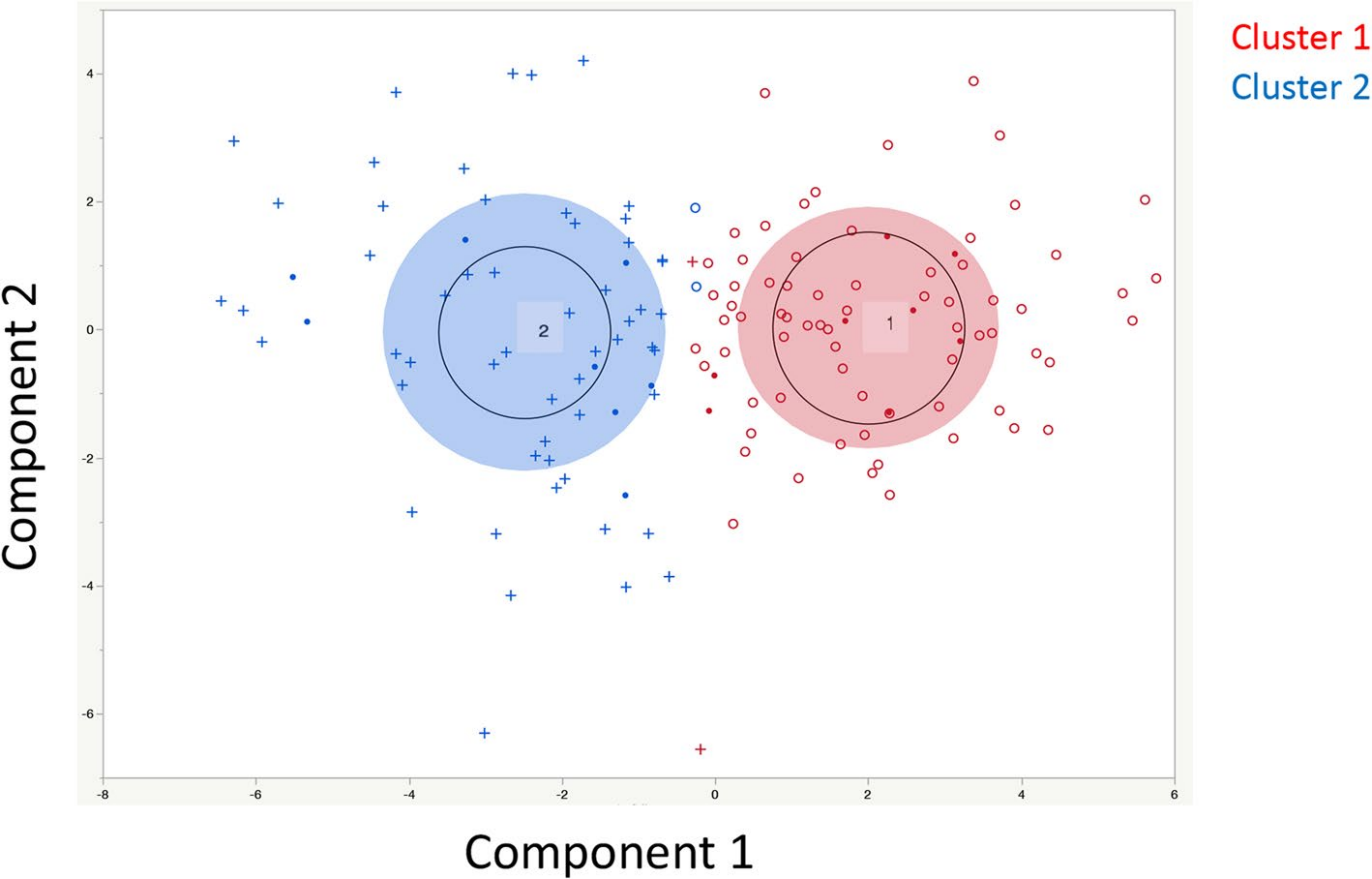
Clustering Results including Clinical Specimen Data. The results were almost the same as those in Fig. 2. The red ‘+’ indicates items classified as cluster 2 in Fig. 2. The blue ‘○’ indicates items classified as cluster 1 in Fig. 2. Only 4 cases had a cluster classification different from the classification in Fig. 2. ‘・’ indicates the results of clinical specimens

**Fig. 5:**
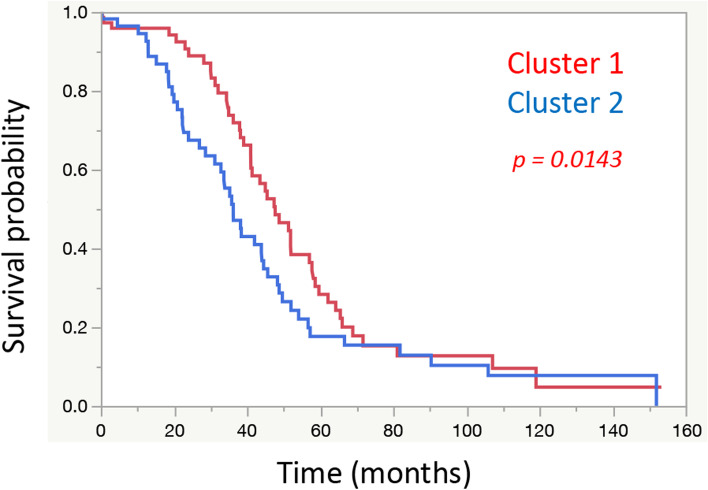
Relationships with Prognosis based on Clustering Results including Clinical Specimen Data (TCGA OV Data). Similar to Fig. 2, among the clusters classified by K means clustering, cluster 2 had a significantly worse prognosis than cluster 1 (*p* = 0.0143)

**Fig. 6:**
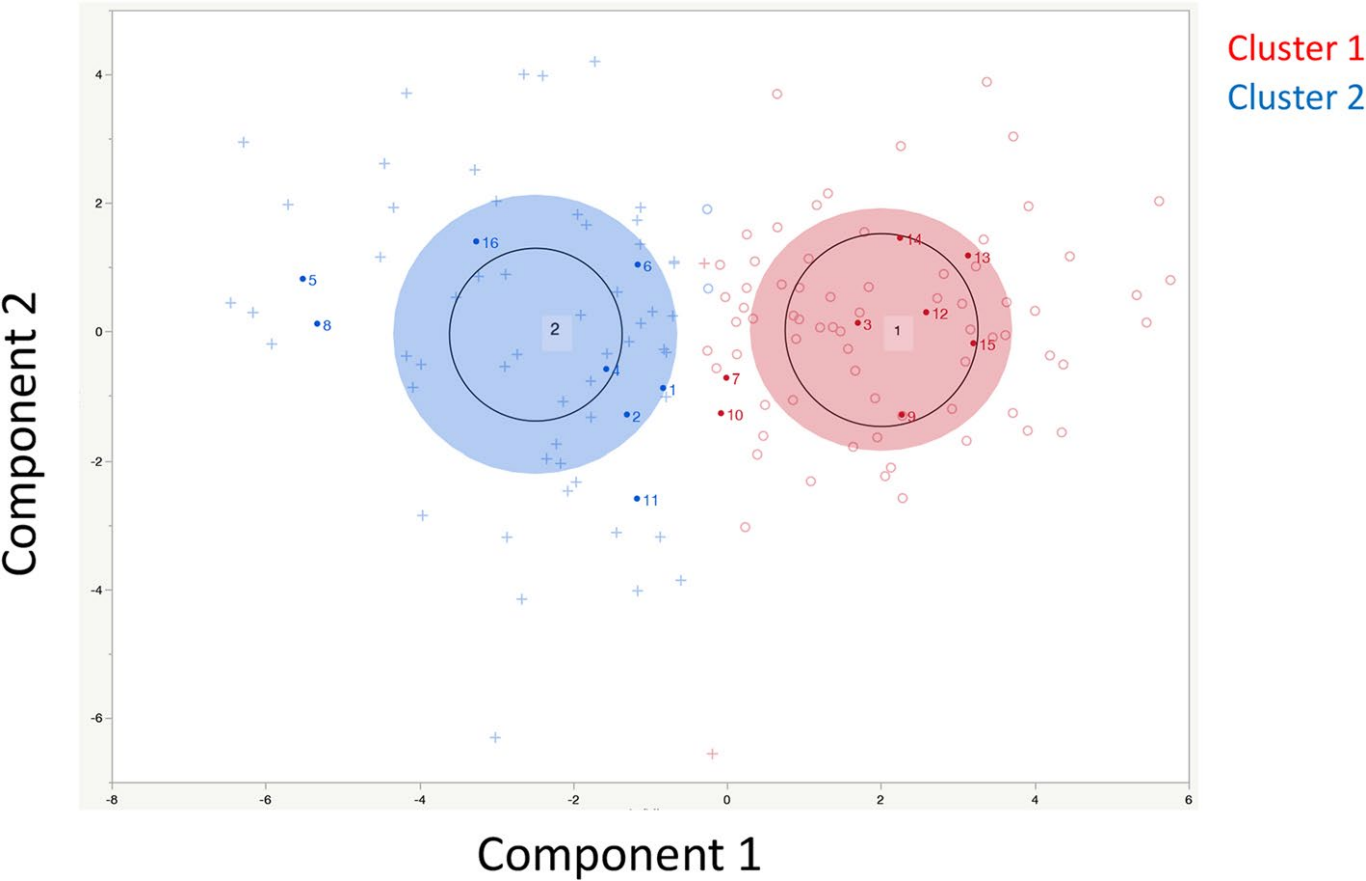
Clustering Results for Clinical Specimens. Each number is the sample number in Table 1

Subsequently, similar clustering including clinical specimen data was performed with the TCGA-OV data. Only 4 cases among 130 cases were classified differently from the abovementioned clustering (Fig. 4). Actually, almost similar results were obtained regarding prognosis (Fig. 5). The relationship between platinum resistance/sensitivity in clinical specimens and this classification is shown in Table 1. In this examination, samples 1-8 are defined as platinum resistant, and samples 9-16 are defined as platinum sensitive. Generally, sample 5 is defined as platinum sensitive because the PFI is 7 months > 6 months. However, the median PFI according to this examination was 12 months. Therefore, sample 5 was defined as platinum resistant in this study. In clusters 1 and 2, cluster 2 was significantly associated with platinum resistance (Fig. 6 and Table 1, χ^2^ test, *p* = 0.0408).

**Fig. 7:**
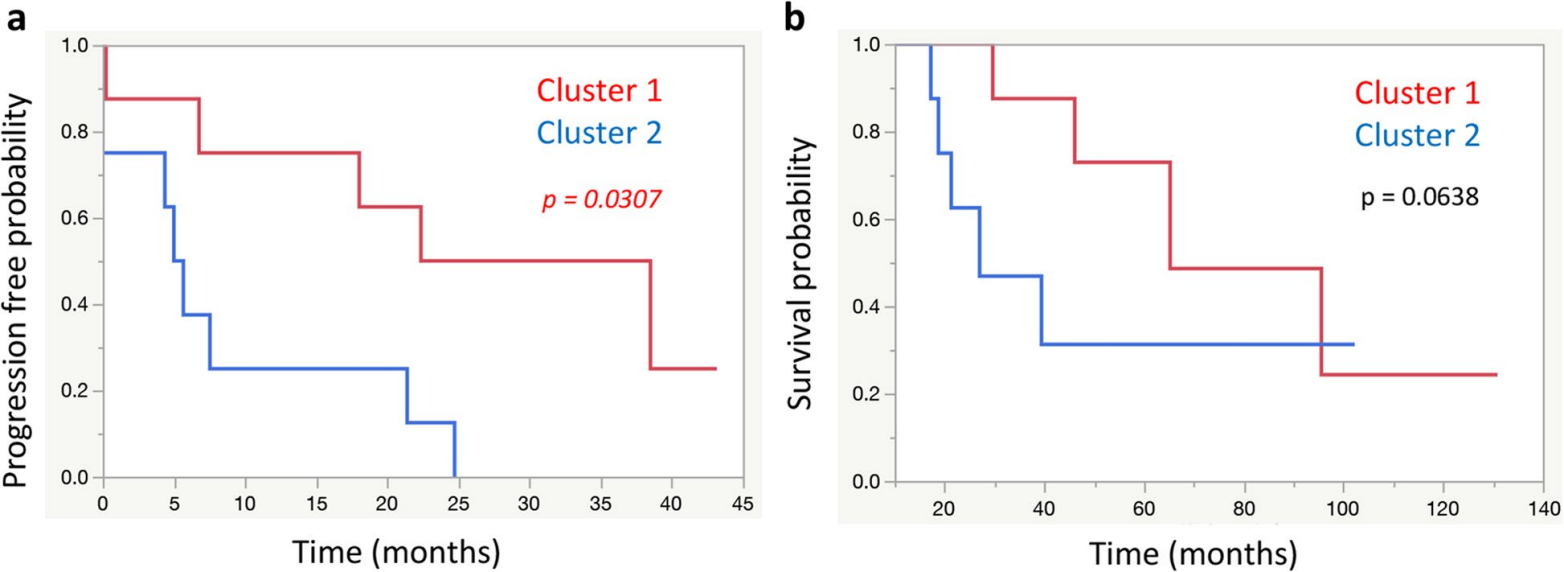
Relationship between Clustering Results including Clinical Specimen Data and Clinical Data. **a** Progression free survival (PFS) based on Clustering Results including Clinical Specimen Data. Cluster 2 had a significantly worse prognosis than cluster 1 in terms of PFS (*p* = 0.0307). **b** Overall survival (OS) based on Clustering Results including Clinical Specimen Data. No significant difference in OS were found between cluster 1 and cluster 2 (*p* = 0.0638)

In this classification, progression-free survival (PFS) after platinum-containing drug administration was examined, and a significant correlation was found (Fig. 7a, *p* = 0.0307). In other words, the group classified as cluster 2 had a significantly shorter PFS than the group classified as cluster 1 in the clinical data. Further, cluster 2 had a worse prognosis tendency with respect to the overall survival (OS) in the clinical data. However, a significant difference was not observed (Fig. 7b, *p* = 0.0638).

## Discussion

By using machine learning including deep learning, in recent years, many studies on applying machine learning in cancer research have been performed [26, 29, 30, 33, 62, 63]. Using machine learning, predicting the prognoses of ovarian cancer patients and the therapeutic effects of platinum-containing drugs can be widely performed [64–72]. In most cases, machine learning from results such as RNA-seq results is first applied [26, 30]. After extracting the gene cluster related to prognosis, the significance is examined using pathway analysis. These methods can accurately predict the prognosis. In fact, when the analysis was performed similarly to this examination, after focusing on the group of genes in references, platinum resistance/sensitivity could be significantly predicted (Table S1, χ^2^ test, *p* = 0.0023). The effects of platinum-containing drugs can be accurately predicted by homologous recombination deficiency (HRD) scores [67, 68]. In these predictions, many pathways are used for prediction, or several cases already awaiting treatment are used [31]. This examination focuses only on gene expression levels related to metabolic pathways and the FAK pathway identified in previous basic experiments and therefore differs from the other examinations. We applied machine learning including neural networks, however, K means clustering was the best to classify groups of platinum resistance/sensitivity in our cases.

In recent years, metabolism in cancer has received considerable attention with the development and popularization of metabolomic analysis [73–76]. Metabolic changes reflect expression levels at the cellular level, and this analysis is closely related to how a cell behaves in the body (that is, whether a cell is highly malignant). In fact, references and self-study cases indicate that targeting the metabolic pathway may have a therapeutic effect on chemotherapy-resistant ovarian cancer [24, 73–76].

The same is true for the FAK pathway. Gene expression related to the FAK pathway was incorporated as a variable in this examination based on reports and previous research indicating that recurrence of ovarian cancer, treatment resistance, and CSCs are related to FAK activity [24, 77–80].

Thus, sensitivity and resistance to platinum-containing drugs can be predicted by focusing on metabolic genes and groups of genes related to FAK activity. As a result, the possibility of predicting the prognosis was shown in this examination. Based on this study, metabolism and the FAK pathway may be potential therapeutic targets in the future. In fact, in the test case, the examination using ovarian clear cell carcinoma cell lines, which are likely to be chemotherapy-resistant, showed a synergistic effect of inhibiting glutamine metabolism and the FAK pathway [24]. However, metabolic activity is determined by the overall balance and not only by high or low levels of each group of genes; thus, suggestions for treatment targeting specific gene expression levels have not been determined from this examination. Also, there are limitations from a selection bias and a small sample size.

We believe CSC-like properties are a useful model which gives us insight into chemo-resistance. Especially, we assumed that investigating CSC-like properties of clear cell carcinoma could give us insight into platinum resistance because most of the patients with ovarian clear cell carcinoma are platinum-resistant. We conducted this study to ensure the results we obtained from our previous *in-vitro* studies. However, there is a possibility that mechanisms of platinum resistance in serous carcinoma is different from that in clear cell carcinoma. In the future, new targets for drug discovery are expected to be found by focusing on metabolism-related genes and FAK activity in treatment-resistant ovarian cancer.

## Supplementary Information

**Additional file 1: Figure S1.** Throughput output of raw and trimmed data.Analyses were successfully performed on all 16 paired-ends samples.

**Additional file 2: Figure S2.** Q30 score of raw and trimmed data. Figure shows the Q30 percentage (% of bases with quality over phred score 30) of each sample’s raw and trimmed data.

**Additional file 3: Figure S3.** Overall read mapping ratio. Trimmed reads are mapped to reference genome with HISAT2 [81]. Figure shows the overall read mapping ratio, the ratio of mapped reads to trimmed reads.

**Additional file 4: Figure S4.** Distribution of the Expression of Each Gene in Each Cluster. (a) The distribution of the expression of each gene in cluster 1. (b) The distribution of the expression of each gene in cluster 2. (c) The mean expression level of each gene in each cluster. Regarding metabolic genes, both high and low expression levels and the overall balance were involved in the metabolic phenotype. Therefore, in this examination, the difference between these 2 groups was unclear

**Additional file 5: Table S1.** Clinical Specimen Data.

## Data Availability

The datasets used and/or analyzed during the current study are available from the corresponding author on reasonable request.

## Abbreviations

FAK: Focal adhesion kinase
FIGO: International Federation of Gynecology and Obstetrics
HGSC: High-grade serous carcinoma
PARP: Poly (ADP-ribose) polymerase
TCGA: The Cancer Genome Atlas
RNA-seq: RNA sequencing
PFI: Platinum free interval
PFS: Progression free survival
OS: Overall survival
