# Supplementary figures and images for: Clinical significance of metabolism-related genes and FAK activity in ovarian high-grade serous carcinoma
Source: BMC Cancer. 2022 Jan 13;22:59. doi: 10.1186/s12885-021-09148-x (PMC8756654; doi:10.1186/s12885-021-09148-x)

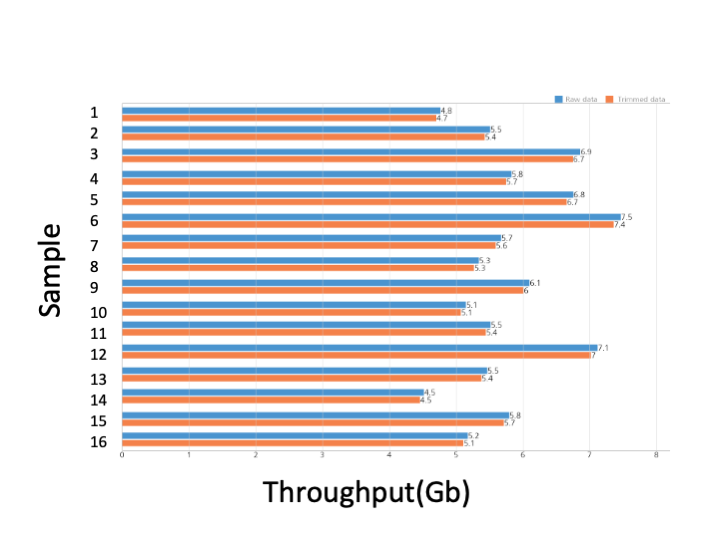

Supplement: Supplementary file 1 — Additional file 1: Figure S1. Throughput output of raw and trimmed data.Analyses were successfully performed on all 16 paired-ends samples. [file 12885_2021_9148_MOESM1_ESM.tiff]

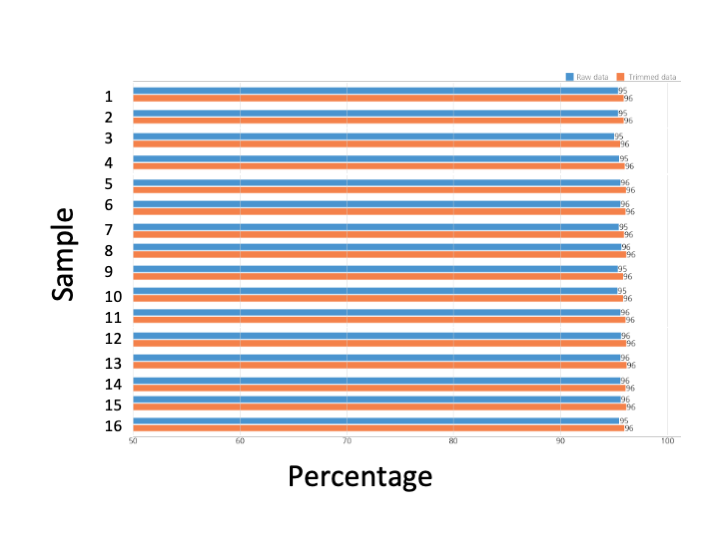

Supplement: Supplementary file 2 — Additional file 2: Figure S2. Q30 score of raw and trimmed data. Figure shows the Q30 percentage (% of bases with quality over phred score 30) of each sample’s raw and trimmed data. [file 12885_2021_9148_MOESM2_ESM.tiff]

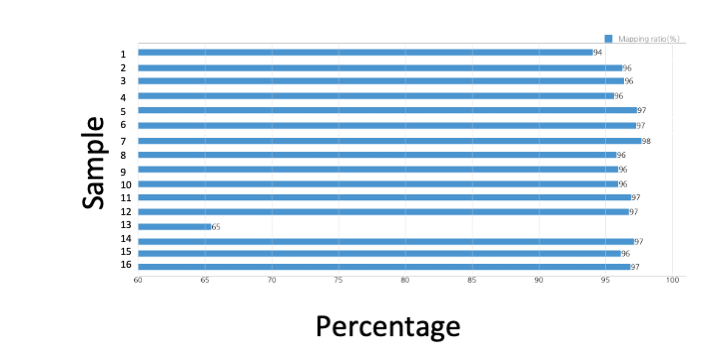

Supplement: Supplementary file 3 — Additional file 3: Figure S3. Overall read mapping ratio. Trimmed reads are mapped to reference genome with HISAT2 [81]. Figure shows the overall read mapping ratio, the ratio of mapped reads to trimmed reads. [file 12885_2021_9148_MOESM3_ESM.tiff]

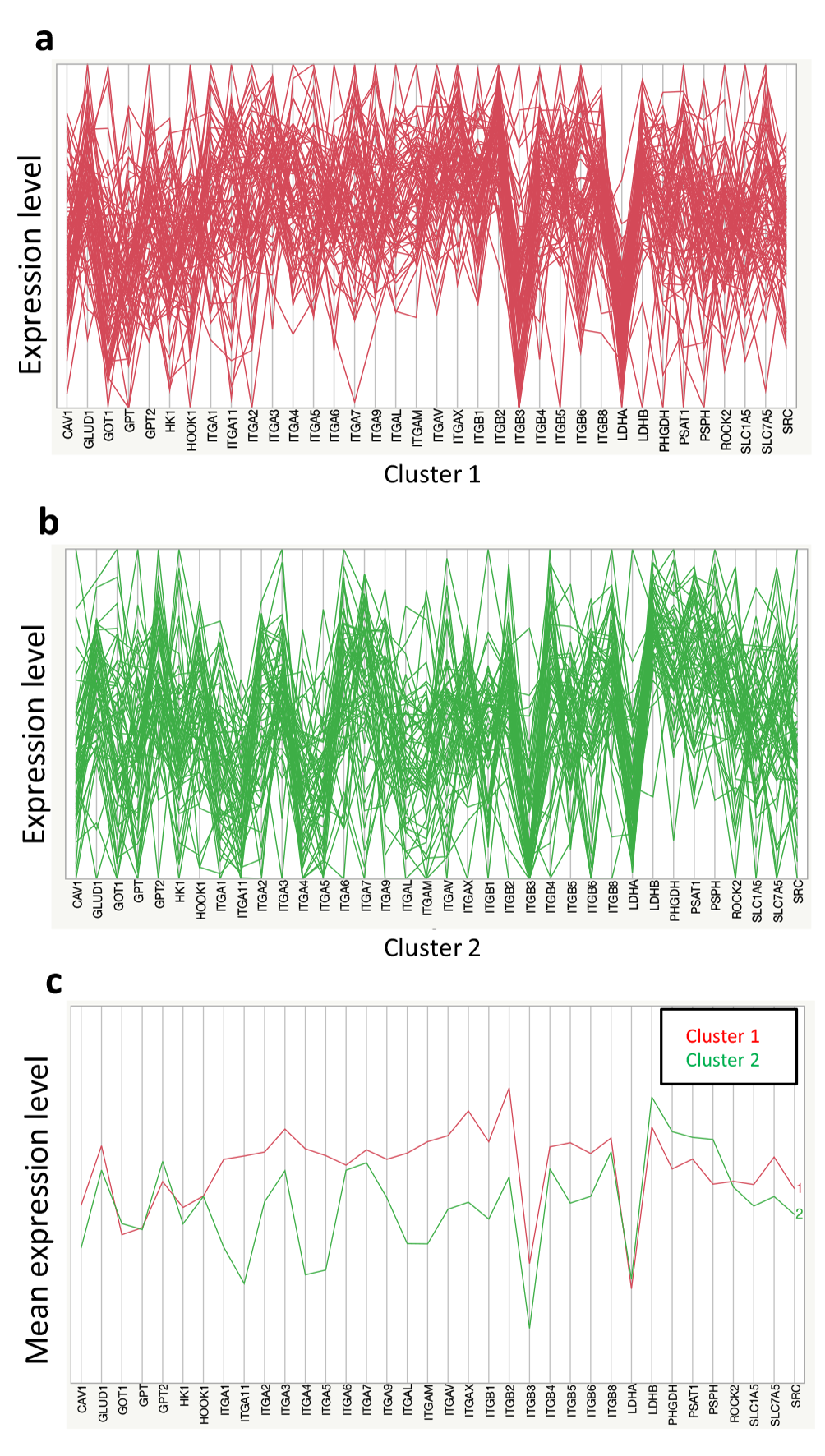

Supplement: Supplementary file 4 — Additional file 4: Figure S4. Distribution of the Expression of Each Gene in Each Cluster. (a) The distribution of the expression of each gene in cluster 1. (b) The distribution of the expression of each gene in cluster 2. (c) The mean expression level of each gene in each cluster. Regarding metabolic genes, both high and low expression levels and the overall balance were involved in the metabolic phenotype. Therefore, in this examination, the difference between these 2 groups was unclear [file 12885_2021_9148_MOESM4_ESM.tif]
