# Supplementary material for: Clinical significance of metabolism-related genes and FAK activity in ovarian high-grade serous carcinoma
Source: BMC Cancer. 2022 Jan 13;22:59. doi: 10.1186/s12885-021-09148-x (PMC8756654; doi:10.1186/s12885-021-09148-x)
Supplement: Supplementary file 5 — Additional file 5: Table S1. Clinical Specimen Data. [file 12885_2021_9148_MOESM5_ESM.docx]

| Sample | Platinum Sensitivity | Clusters (This Examination) | Clusters (From References) |
| --- | --- | --- | --- |
| 1 | R | 2 | 2 |
| 2 | R | 2 | 2 |
| 3 | R | 1 | 2 |
| 4 | R | 2 | 2 |
| 5 | R | 2 | 2 |
| 6 | R | 2 | 2 |
| 7 | R | 1 | 2 |
| 8 | R | 2 | 2 |
| 9 | S | 1 | 2 |
| 10 | S | 1 | 1 |
| 11 | S | 2 | 2 |
| 12 | S | 1 | 1 |
| 13 | S | 1 | 1 |
| 14 | S | 1 | 1 |
| 15 | S | 1 | 1 |
| 16 | S | 2 | 2 |

**Table S1. Clinical Specimen Data.**

Results from analysing similar clusters using genes related to platinum sensitivity shown in a previous report (31). The classification results using genes in the references showed a significant correlation with platinum sensitivity (χ^2^ test, p = 0.0023).

R, platinum resistance, S, platinum sensitive.
